# The impact of HIV-1 infection and exposure on natural killer (NK) cell phenotype in Kenyan infants during the first year of life

**DOI:** 10.3389/fimmu.2012.00399

**Published:** 2012-12-31

**Authors:** Jennifer A. Slyker, Barbara Lohman-Payne, Grace C. John-Stewart, Tao Dong, Dorothy Mbori-Ngacha, Kenneth Tapia, Ann Atzberger, Stephen Taylor, Sarah L. Rowland-Jones, Catherine A. Blish

**Affiliations:** ^1^Department of Global Health, University of WashingtonSeattle, WA, USA; ^2^MRC Human Immunology Unit, Weatherall Institute of Molecular Medicine, Oxford UniversityOxford, UK; ^3^Department of Medicine, University of WashingtonSeattle, WA, USA; ^4^Department of Paediatrics and Child Health, School of Medicine, University of NairobiNairobi, Kenya; ^5^Department of Epidemiology and Department of Pediatrics, University of WashingtonSeattle, WA, USA; ^6^MRC Molecular Haematology Unit, Weatherall Institute of Molecular Medicine, Oxford UniversityOxford, UK; ^7^Computational Biology Research Group, Weatherall Institute of Molecular Medicine, Oxford UniversityOxford, UK; ^8^Department of Medicine and Stanford Immunology, Stanford University School of MedicineStanford, CA, USA

**Keywords:** NK cell, HIV-1, infancy, mother-to-child transmission, age, exposure, immune activation, cord blood

## Abstract

Natural killer (NK) cells play an important role in the containment of HIV replication during primary infection, though their functions are impaired during chronic HIV infection. Infants experience more rapid HIV disease progression than adults, but contributions of infant NK cells to containing HIV infection are unknown. The aim of this study was to determine the impact of HIV infection on infant NK cell phenotype by evaluating samples and data from a cohort study of women and their infants, conducted in Nairobi, Kenya between 1999 and 2003. The percentage and phenotype of NK cells was evaluated longitudinally by multi-parameter flow cytometry over the first year of life in HIV-infected (HIV+, = 16), HIV-exposed uninfected (HIV-EU, *n* = 6), and healthy unexposed controls (HIV–, *n* = 4). At birth, NK subset distributions based on expression of CD56 and CD16 did not differ between HIV+, HIV-EU, or HIV– infants. However, HIV infection was associated with a subsequent decline in NK cells as a percentage of total lymphocytes (*p* < 0.001), and an expanding proportion of CD56-CD16+ NK cells (*p* < 0.001). Activated CD38^bright^CD69+ NK cells were more frequent in the HIV+ infants, followed by HIV-EU and HIV- infants, in both CD56^dim^ (*p* = 0.005) and CD56^bright^ compartments (*p* = 0.03). HIV infection and exposure was also associated with a significant decline in the percentage of perforin-expressing NK cells in the CD56^dim^ compartment over the first year of life, with HIV+ infants losing approximately 2.5% (*p* < 0.001) and HIV-EU infants losing 3.0% (*p* = 0.01) of perforin+ cells per month. Thus, infant HIV infection is associated with alterations in NK cell subsets, activation, and cytolytic potential that could contribute to their poor control over HIV infection. Furthermore, exposure to HIV infection in infants who escaped infection is also associated with alterations in NK cells that may contribute to the reduced ability to fight infections that is observed in HIV-EU infants.

## Introduction

Natural killer (NK) cells are innate lymphocytes that can respond quickly, without prior sensitization, to eliminate virus-infected cells, making them critical in the early response to viral infection (Cerwenka and Lanier, 2001; Lanier, 2008; Biron, 2010; Vivier et al., 2011). Recent evidence from studies conducted in adults have highlighted a significant role for NK cells in the control and prevention of HIV-1 infection (Cerwenka and Lanier, 2001; Fauci et al., 2005; Lanier, 2008; Alter and Altfeld, 2009; Biron, 2010; Tomescu et al., 2011; Vivier et al., 2011). Although innate immunity plays a key role in neonatal defenses as the adaptive immune response develops, very few studies have examined role of NK cells in the defense against infant HIV acquisition, or the containment of HIV after infection (Bernstein et al., 2004; Azzoni et al., 2005; Ballan et al., 2007).

Infant HIV infection is characterized by very poor containment of HIV replication; both peak and set point viral load are often >1 log higher than in adults, and are higher among infants infected peripartum compared to those infected later via breast milk (Richardson et al., 2003). These high infant viral loads are associated with a very high risk of mortality in the absence of antiretroviral therapy (ART) (Blanche et al., 1990; De Rossi et al., 1996; Dickover et al., 1998; Luzuriaga et al., 1999; Obimbo et al., 2004). In the setting of ART, infants attain viral suppression more slowly than adults and many fail to achieve suppression after >24 months of therapy (Penazzato et al., 2012). In adults, HIV-specific T cell responses contribute to the rapid decline in HIV viral load during the first weeks of primary infection (Walker et al., 1991; Koup et al., 1994; Bollinger et al., 1996; Hess et al., 2004); in infants, this pattern is often absent, and is attributed to a combination of lower HIV-specific T cell numbers, altered T cell phenotype, fewer polyfunctional cells, and early emergence of CTL escape mutations (Goulder et al., 2001; Hansasuta and Rowland-Jones, 2001).

In this setting of sub-optimal T cell responses, NK cells could potentially play a very important role in infant primary HIV infection. While NK cells appear to be critical for the early containment of HIV infection in adults, their phenotype and functions were found to be dramatically altered following HIV acquisition. Acute HIV infection is characterized by an early expansion of NK cells, particularly of the CD3-CD56^dim^ subset (Hu et al., 1995; Alter et al., 2007). Although there are reports of increased NK cell activity during viremic HIV infection (Alter et al., 2004; Eller et al., 2009), during the chronic phase of HIV infection, NK cell numbers are low in the absence of ART (Azzoni et al., 2002; Alter et al., 2005; Mantegani et al., 2010), and most have diminished ability to perform cytolysis and secrete cytokines (Cai et al., 1990; Brenner et al., 1993; Ullum et al., 1995, 1999; Azzoni et al., 2002; De Maria et al., 2003; Mavilio et al., 2003, 2006; Fogli et al., 2004; Barker et al., 2007; Brunetta et al., 2009; Mantegani et al., 2010). Chronic HIV infection also leads to alterations in the NK cell subset distribution, with a decline in the proportion of CD56+ NK cells and a dramatic expansion of CD56-CD16+ NK cells (reviewed in Alter and Altfeld, 2009). These alterations in phenotype and percentage may be partially reversed by administration of ART, implicating active viral replication in inducing these changes (Mavilio et al., 2003, 2006; Alter and Altfeld, 2009). The CD56-CD16+ NK cells lack most effector functions and appear to be anergic (Alter and Altfeld, 2009). Chronic HIV infection is also associated with phenotypic changes in NK cells, including increased expression of activation markers and reduced expression of natural cytotoxicity receptors (De Maria et al., 2003; Fogli et al., 2004; Mantegani et al., 2010).

To our knowledge, only two studies have directly examined NK cells in children who were HIV-infected during infancy. In one study, HIV-infected children (median age 11.1 years) had a sustained depletion in NK cells that only partially recovered with ART (Azzoni et al., 2005). In the second study, there were no significant differences in the NK cell subset distribution between HIV-infected children receiving ART (median age 8.2 years) and HIV-exposed uninfected children (median age 5.5) children (Ballan et al., 2007). However, the NK cells from the HIV-infected children had some phenotypic differences and decreased cytolytic activity in comparison to the NK cells from the HIV-exposed uninfected children (Ballan et al., 2007). These data indicate that chronic HIV infection in children can impair NK cell function, but these studies were subject to survivor bias, and they were not able to examine early events in infancy, when NK cells might be particularly important to control HIV infection.

Since the early response to HIV infection is critical in determining the subsequent disease course (Mellors et al., 1996; Lifson et al., 1997; Gandhi and Walker, 2002), it is important to understand the dynamics of early infection in infants. Our prior studies in this cohort demonstrated that HIV-infected neonates could generate CD8+ T cell responses early in life, but that these were not associated with improved clinical outcomes (Lohman et al., 2005). Furthermore, infant T cells were persistently activated and vulnerable to apoptosis (Slyker et al., 2011), which could explain in part their reduced effectiveness in containing viremia. We hypothesized that age and HIV exposure would also affect NK cell phenotype during the first year of life. In the current study, we present a longitudinal analysis of the percentage and phenotype of NK cells over the first year of life in HIV-infected, HIV-exposed uninfected, and healthy HIV-unexposed infants, and examine the relationship between NK cell phenotype and HIV viral load.

## Materials and methods

### Participants and specimen collection

Study protocols were performed in accordance with all relevant institutional and national guidelines and were approved by the Ethics Review Committees of Kenyatta National Hospital and University of Washington. Frozen PBMC specimens were utilized from an historic cohort study conducted in Nairobi, Kenya from 1999 to 2003 as previously described (Obimbo et al., 2004; Lohman et al., 2005; John-Stewart et al., 2009; Slyker et al., 2011). Between 1999 and 2003, HIV-1 infected women were recruited during pregnancy and provided with short-course zidovudine for prevention of HIV-1 transmission (Shaffer et al., 1999). Infants who acquired HIV infection were followed for 2 years following delivery; infants that were HIV-exposed but uninfected were followed for 12 months. Serial infant blood specimens were collected at delivery, and 1, 3, 6, 9, 12, 15, 18, 21, and 24 months of age. A set of HIV-uninfected women and their children (HIV-unexposed) were additionally enrolled as controls; these women were recruited from the same antenatal clinics as the HIV-infected women. Healthy, HIV-unexposed infants were followed for six months, with blood collection at delivery and 6 months of age only.

Infant peripheral blood and cord blood was collected into EDTA tubes and separated into plasma and PBMC using density-gradient centrifugation over Ficoll (GE Healthcare, Chicago, IL, USA). Plasma was frozen at −70°C; PBMC were cryopreserved in fetal calf serum (FCS) supplemented with 10% DMSO freezing media and stored in liquid nitrogen. Between 2 and 10 million PBMC were frozen in each vial.

### Immunophenotyping NK cells

Flow cytometry experiments were conducted on frozen specimens between 2005 and 2006. A sample of infants was selected for immunology assays based upon the availability of cryopreserved PBMC specimens. The median viability of thawed PBMCs during this time was 72.4% (interquartile range 60.0–80.5%). As previously described, cells were stained with panels of antibodies to identify activated and apoptosis-vulnerable T cells (Slyker et al., 2011, 2012). Cells were thawed in FCS, washed twice with PBS plus 0.5% BSA and 0.5 mM EDTA (PBE), and stained with CD3-Pacific Blue (UCHT1, Dakocytomation, Angel Drove, UK), CD4-APC-Cy7 (RPA-T4, BD Pharmingen, Oxford, UK), CD8-PE-Cy7 (RPA-T8, Pharmingen), CD56-PE-Cy5 (N901 (NKH-1), Beckman Coulter, High Wycombe, UK), CD16-APC-Cy7 (3G8, Pharmingen), CD69-FITC (FN50, Dakocytomation), CD38-PE (AT13/5, Serotec, Oxford, UK), HLA-DR-APC (TU36, Pharmingen), CD57-FITC (TB01, Serotec), CD71-APC (M-A712, Pharmingen), CD95-APC (DX2, Pharmingen), Bcl-2-FITC (124, Dakocytomation), Perforin-PE (27-35, Pharmingen), CD27-APC (0323, eBioscience, San Diego, CA, USA), CD28-FITC (CD28.1, Dakocytomation), CCR7-PE (FAB197, R&D Systems, Minneapolis, MN, USA) and CD45RA-APC (HI-100, Pharmingen). Cells were stained for 20 min in the dark, and washed twice with PBE and resuspended in CellFix solution (Pharmingen). In all assays, fixed and stained cells were stored overnight at 4°C and analyzed the next morning in Nairobi, Kenya.

Cells were acquired on a Cyan ADP or LX instrument using Summit software (Dako Cytomation, Angel Drove, UK) and data were analyzed with FlowJo software v8.8.7 (Treestar, Inc., Olten, Switzerland). Isotype controls were used to set gates for phenotypic markers. In order to minimize intra-patient variability, all time-points for a single infant were thawed, stained, and analyzed at the same time. Gates were set using the earliest infant sample available, then applied to all subsequent time-points. This enabled us to track migration of cells between quadrants and gates as a function of time.

### HIV-1 diagnosis and quantification

HIV-1 RNA viral loads were measured from cryopreserved plasma using the Gen-Probe assay (Emery et al., 2000) and dried blood spots were used for DNA PCR of the HIV-*gag* gene as previously described (Panteleeff et al., 1999). Infant HIV-1 infection was defined by two consecutive positive results as measured by either HIV-1 RNA or DNA. Infants with HIV-1 detection in the first 48 h were considered *in utero* transmissions; those with infection between 48 h and 1 month were considered peripartum transmissions. No infants with HIV-1 acquisition after 1 month were included in the current study.

### Statistical analyses

Stata SE v11 (Stata Corp. College Station, TX, USA) was used for all statistical analyses. All tests were two-tailed. NK subset distributions were compared between infant groups at birth; Kruskal–Wallis tests were used to assess for overall differences, then pairwise Mann–Whitney U-tests were used to assess differences between groups.

Linear mixed models (LMM) were used to assess whether the % of NK cells (gated on lymphocytes), or NK cell subsets (%CD56^bright^, %CD56+CD16+, and %CD56-CD16+, gated on total NK cells), changed during the first year of life. Models were run separately for each HIV infection group.

LMM was also used to compare the phenotype of CD56^bright^ to the phenotype of CD56^dim^ cells within individual infants. The [CD56subset] term indicated whether the cells were gated on CD56^bright^ = 1 or CD56^dim^ = 0. For each phenotype, an LMM was fitted that included [CD56subset] and [time]. LMM models were run separately for HIV-infected, HIV-exposed uninfected, and HIV-unexposed infants. Point estimates (mean differences) for each phenotype were similar for infants in each group, so the final data presented show all infants as a single group.

For all phenotypic analyses comparing HIV infection groups over time, data were analyzed separately for CD56^bright^ and CD56^dim^ subsets. To determine the effect of HIV status on NK phenotype, we created a variable scaled by increasing HIV exposure [HIV status]: HIV-unexposed = 0, HIV-exposed uninfected = 1, HIV-infected = 2. LMM were used to assess whether the percentage of cells of each phenotype (activated, apoptosis-vulnerable, etc.) changed over time. For each phenotype, a model was fit that included: a main term [HIVstatus] for HIV exposure, age in months [time], and an interaction term for [HIVstatus] × [time]. When the *p*-value for the interaction term was >0.05, the slopes for HIV-infected, HIV-exposed uninfected, and HIV-unexposed infants were considered equivalent, the interaction term was removed from the model, and we report the overall combined slope. For each phenotype, we also present the beta-coefficient for the HIV status term, which estimates the mean difference in percentage of cells incrementally between the categories of HIV-unexposed, HIV-exposed uninfected, and HIV-infected infants. We additionally report individual slopes and for HIV-infected, HIV-exposed uninfected, and HIV-unexposed infants. We also used LMM to examine the relationship between percentages of activated and apoptosis-vulnerable cells in the CD56^bright^ subset, with [%CD38^bright^HLA-DR+] and [time] as main terms, including all of the infants.

In HIV-infected infants, LMM models were used to assess whether the % of cells of each phenotype were associated with log_10_ HIV RNA viral load at the concurrent time-point. Models were also adjusted for time (age in months). For each phenotype, an LMM was fitted that included [log_10_ HIV RNA viral load] and [time].

All LMM models described above included random intercepts and slopes and unstructured covariance matrices. All tests were two-tailed, using alpha = 0.05.

## Results

### Study participants and follow-up

Samples from 26 infants who were enrolled in a historical cohort study of mother to child transmission of HIV-1 were evaluated to determine the effects of HIV-1 infection and exposure on NK cell percentage and phenotype (Table 1). Sixteen infants were HIV-infected, and of those, 6 were infected *in utero* and 10 were infected in the first month of life (peripartum). The HIV-infected infants had a mean peak viral load of 7.1 log_10_ copies/mL and 56% died before two years of age. HIV-exposed uninfected infants (*n* = 6), and healthy control infants (HIV-unexposed, *n* = 4) all survived follow-up. Samples from a median of 5 time points from HIV-infected and HIV-exposed uninfected infants were evaluated; samples from HIV-unexposed infants were tested at two time points, birth and at 6 months of age.

**Table 1:** **Characteristics of study subjects**.

**Characteristic**	**HIV+^a^**	**HIV-EU^b^**	**HIV-^c^**
Number of infants	16	6	4
Infected *in utero^d^*	6	–	–
Infected *peripartum^e^*	10	–	–
Median follow-up time in months (IQR)	12 (7.5–21)	12 (12–12)	6 (3–6)
Median number of study visits tested	5 (3–5.5)	5 (3–6)	2 (1.5–2)
Mean (±SD) peak plasma HIV-1 viral load (log_10_ copies/mL)	7.1 (±0.64)	NA	NA
Deaths	9 (56%)	0	0

a *HIV+: HIV-infected*.

b *HIV-EU: HIV-exposed uninfected*.

c *HIV-: Healthy HIV-unexposed control infants*.

d *In utero infection was determined on the basis of HIV DNA or RNA detected <48 h of birth*.

e *Peripartum HIV infection was defined as HIV DNA or RNA negative at <48 h and positive at 1 month*.

### Distribution of NK cell subsets in HIV-infected, HIV-exposed uninfected, and healthy infants at birth

**Figure 1:**
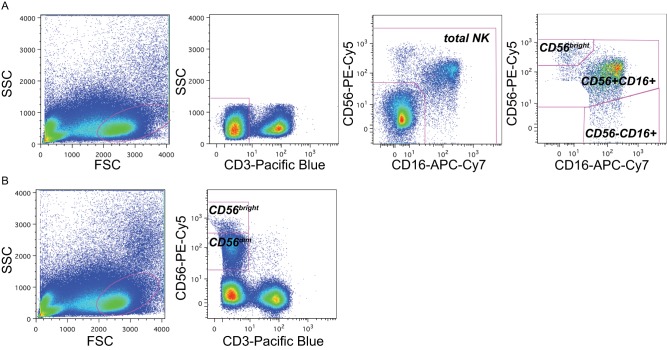
**Gating strategy to identify NK subsets.** Representative flow cytometry plots from the cord blood of one representative subject within the cohort (subject B1-081) to demonstrate gating strategy. **(A)** In panels that included CD16 as a maker, NK cell subsets were identified by sequentially gating on lymphocytes, then CD3-cells, then cells expressing CD16 and/or CD56. NK cell subsets were identified as CD56^bright^ (upper right gate), CD56+CD16+, and CD56-CD16+. **(B)** In panels without CD16 staining, NK cells were identified as CD3-lymphocytes that were either bright (CD56^bright^) or dim (CD56^dim^) for CD56 staining; the CD56-population could not be distinguished.

The major NK cell subsets were defined by excluding granulated cells, dead cells, and debris by size and granularity (Figure 1A). Next, CD3 expression was used to exclude T cells, and NK cells were identified on the basis of expression of CD56 and/or CD16. Three distinct subsets comprised the total NK cell pool: CD56^bright^ cells, CD56+CD16+ cells, and CD56-CD16+ cells (Figure 1A). In staining panels in which CD16 was not available, CD56- NK cells could not be identified, but NK cells were identified as small CD3-lymphocytes that were characterized as CD56^dim^ and CD56^bright^ NK cells (Figure 1B).

At birth, NK cells comprised approximately 13% of lymphocytes (Table 2), consistent with previous studies (Luevano et al., 2012). The majority of infant NK cells were CD56^dim^CD16+ (median of 75%), and the minority were CD56^bright^ (median of 6.0%). CD56-CD16+ cells, previously observed both in cord blood and during chronic HIV-1 infection (Alter et al., 2005; Verneris and Miller, 2009; Luevano et al., 2012), were detected in all groups at birth, and comprised 18% of total NK cells. We detected no significant differences in subset distributions between infants categorized by HIV status or exposure at birth (*p* > 0.05 for each comparison).

**Table 2:** **Distribution of NK cell subsets in HIV-unexposed, HIV-exposed uninfected, and HIV-infected infants at birth**.

	**All infants (*n* = 22)**	**HIV+^a^ (*n* = 5)**	**HIV-EU (*n* = 5)**	**HIV- (*n* = 4)**	**Kruskal–Wallis *P*-value^b^**
Total NK^c^	13 (7.3–19)	16 (7.3–35)	11 (7.3–13)	17 (10–21)	0.6
CD56^bright^	6.0 (4.6–8.3)	8.3 (5.9–9.5)	6.1 (5.5–6.3)	4.9 (3.3–10)	0.8
CD56+CD16_+_	75 (68–84)	75 (70–77)	79 (75–80)	85 (76–86)	0.4
CD56-CD16_+_	18 (14–21)	19 (14–21)	18 (14–19)	12 (8.8–15)	0.2

a *HIV+, HIV-infected; HIV-EU, HIV-exposed uninfected; HIV-, healthy HIV-unexposed control infants*.

b *All pair-wise tests between infants grouped by HIV-1 infection, p > 0.05*.

c *Total NK = Percentage of CD3-CD56+ and CD3-CD16+ cells in lymphocyte gate. All other subsets as % of total NK population. Due to rounding percentages will not add exactly to 100%*.

### Changes in the distribution of NK cell subsets over time

**Figure 2:**
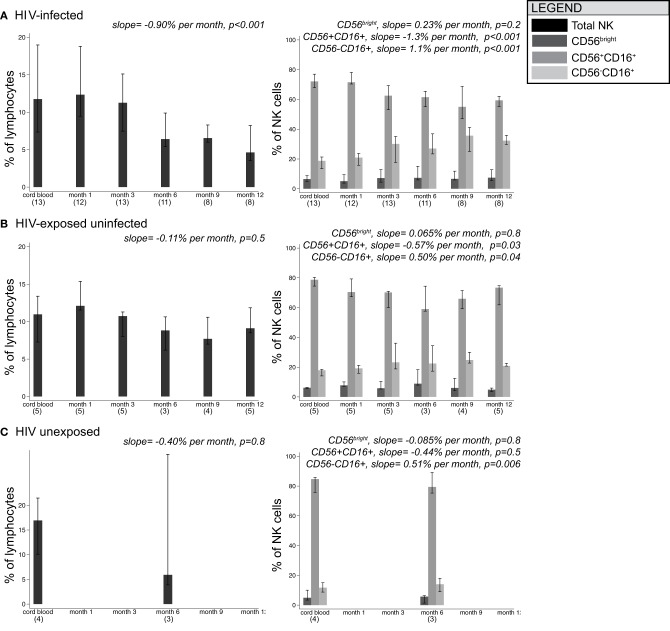
**Median frequencies of NK cell subsets in infants grouped by HIV-1 infection/exposure during the first year of life.** Bars show median, with 25th and 75th percentiles for HIV-infected infants **(A)**, HIV-exposed uninfected infants **(B)**, and HIV-unexposed infants **(C)**. (*n*) indicates number of infant specimens tested at each time point. Linear mixed models were used to test for the effect of age on percentage of cells; beta coefficients and *p*-values for slope are provided.

Figure 2 shows changes in the distribution of NK cell subsets over time in HIV-infected, HIV-exposed uninfected, and HIV-unexposed infants. In HIV-infected infants, the total NK cell percentage decreased over time at a rate of 0.90% per month (*p* < 0.001). The total NK cell percentage did not change significantly over time in HIV-exposed uninfected (−0.11% per month, *p* = 0.5) and HIV-unexposed infants (0.16% per month, *p* = 0.8).

As a percentage of total NK cells, CD56^bright^ cells remained stable over time in all groups of infants. In HIV-infected infants, CD56+CD16+ cells declined at a rate of –1.3% per month (*p* < 0.001) in parallel with an increase in CD56-CD16+ cells at a rate of +1.1% per month (*p* < 0.001), consistent with the current hypothesis that CD56+CD16+ cells lose CD56 expression in response to persistent stimulation during chronic viral infection (Alter et al., 2005; Björkström et al., 2010). Although we had limited power to detect time trends in HIV-exposed uninfected and HIV-unexposed infants, we observed similar trends in the changes in subset distribution over time.

### Phenotypic composition of the NK cell subsets

The activation and differentiation status of NK cells was assessed by the expression of several surface and intracellular markers. Regardless of infection status, NK cells were uniformly CD38+ throughout most of infancy, expressed CD45RA and lacked expression of CD28 and CCR7 (data not shown). NK cells were defined as early activated (CD38+CD69+), activated (CD38^bright^HLA-DR+), vulnerable to apoptosis (CD95+Bcl-2^low^), proliferating (CD71+CD57–), terminally differentiated (CD71-CD57+) or potentially cytolytic (perforin+). The phenotype of cells differed remarkably between CD56^bright^ and CD56^dim^ NK subsets in all infants regardless of their infection status. Table 3 shows the mean difference in % of cells of each phenotype between the CD56^bright^ and CD56^dim^ NK subsets. For example, at any given time-point, there were 4.1% fewer CD38^bright^CD69+ cells in the CD56^bright^ compared to the CD56^dim^ subset. Cells of the CD56^bright^ subset contained the majority of cells that were proliferating (CD71^+^CD57^−^) and were vulnerable to apoptosis (CD95^+^Bcl-2^dim^), while the CD56^dim^ subset contained the majority of cells that expressed the early activation marker CD69 (CD38^bright^CD69^+^), were terminally differentiated (CD71^−^CD57^+^), and had cytolytic potential (perforin+). When stratified by HIV status, we saw a very similar pattern of differences between NK subsets; despite the reduced statistical power, point estimates were very similar between HIV-infected and HIV-exposed uninfected (data not shown).

**Table 3:** **Phenotypic composition of CD56^bright^ and CD56^dim^ subsets**.

**Phenotype**	**% within CD56^bright^ subset at baseline^a^**	**% within CD56^dim^ subset at baseline^a^**	**Mean difference between CD56^bright^ and CD56^dim^ subsets**	***P*-value**
Early activated (CD38^bright^CD69^+^)	10% (6.5, 14)	14% (11, 18)	−4.1 [−6.3, −1.9]	<0.001
Activated (CD38^bright^HLA-DR^+^)	23% (15, 30)	14% (6.0, 21)	+9.5 [4.9, 14]	<0.001
Vulnerable (CD95^+^Bcl-2^dim/-^)	18% (12, 24)	7.3% (1.4, 13)	+11 [7.0, 14]	<0.001
Proliferating (CD71^+^CD57^−^)	46% (40, 53)	2.3% (−4.7, 8.7)	+45 [41, 49]	<0.001
Terminally differentiated (CD71^−^CD57^+^)	2.8% (−0.39, 6.1)	13% (9.9, 16)	−10 [−14, −6.6]	<0.001
Potentially cytolytic (perforin+)	33% (20, 46)	60% (47, 72)	−28 [−36, −20]	<0.001

*Linear mixed models (LMM) were used to conduct within-subject paired comparisons for percent of activated, vulnerable, proliferating, differentiated, and potentially cytotoxic cells gated on CD56^bright^ vs. CD56^dim^ cells*.

a *Baseline (birth) percentages are estimated by LMM model at time = 0 (y-intercept). Analysis uses all time-points from all infants (*n* = *26*). The median number of cells acquired was 446 cells (IQR = 207–980) for the CD56^bright^ and 6647 cells (IQR = 2791–12,778) for the CD56^dim^ subset*.

### NK cell activation during acute HIV infection

**Figure 3:**
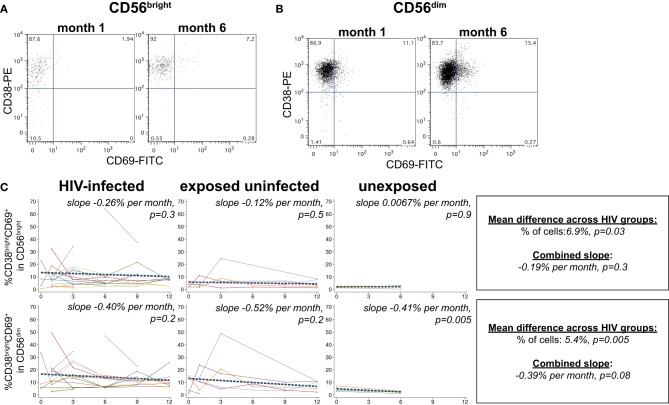
**CD38^bright^HLA-CD69+ NK cells.** Representative flow cytometry plots from NK cell subsets showing identification of CD38^bright^CD69+ cells in **(A)** CD56^bright^ and **(B)** CD56^dim^ subsets at 1 and 6 months of age an HIV-infected infant (B1-093). **(C)** Plots show individual infant trajectories as connected lines of different colors, with overlaid linear mixed models (LMM). LMM were used to determine whether slope (change in % of cells/month) was affected by HIV infection status; where slopes are not different, the slope representing the combined data of all infants is shown to the box on the right. The mean difference across HIV groups is derived from the beta coefficient for [HIV status] as described in the Materials and Methods, and represents the % increase in cells per HIV category from HIV-unexposed to HIV-exposed uninfected to HIV-infected. Slopes for each HIV infection group are also presented separately within each graph.

**Figure 4:**
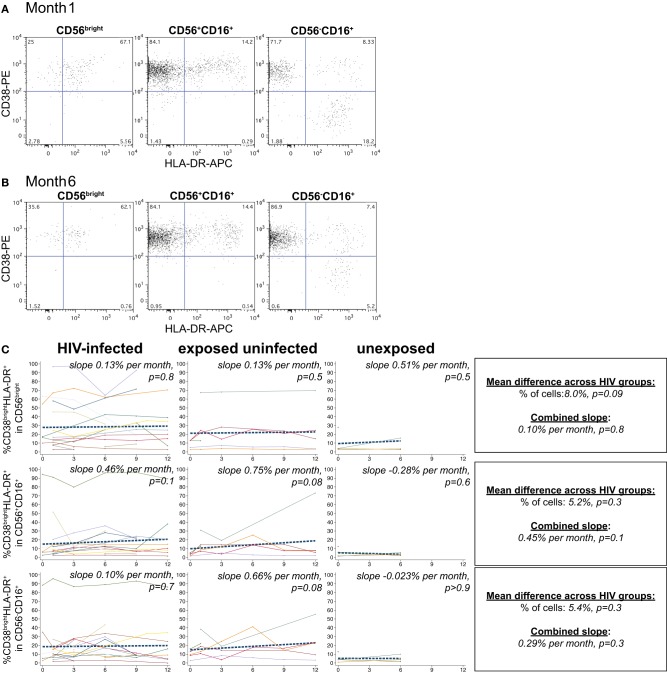
**CD38^bright^HLA-DR+ NK cells.** Representative flow cytometry plots from NK cell subsets showing identification of CD38^bright^HLA-DR+ cells at **(A)** 1 month and **(B)** 6 months of age an HIV-infected infant (B1-093). **(C)** Plots show individual infant trajectories as connected lines of different colors, with overlaid linear mixed models (LMM). LMM were used to determine whether slope (change in % of cells/month) was affected by HIV infection status; where slopes are not different, the slope representing the combined data of all infants is shown to the box on the right. The mean difference across HIV groups is derived from the beta coefficient for [HIV status] as described in the Materials and Methods, and represents the % increase in cells per HIV category from HIV-unexposed to HIV-exposed uninfected to HIV-infected. Slopes for each HIV infection group are also presented separately within each graph.

NK cells expressing the activation markers CD69 and HLA-DR were detected among all NK subsets (Figures 3A,B and 4A,B). In the CD56^bright^ subset, the mean percentage of CD38^bright^CD69^+^ cells in HIV-infected infants was 6.9% higher than HIV-exposed uninfected and 13.8% higher than HIV-unexposed infants (*p* = 0.03, Figure 3C). A similar pattern was observed in the CD56^dim^ subset, the mean percentage of CD38^bright^CD69^+^ cells in HIV-infected infants was 5.4% higher than HIV-exposed uninfected and 10.8% higher than HIV-unexposed infants (*p* = 0.005). Though there was a downward trend in the percentage of early activated NK cells in many infants over time, this was statistically significant only in the HIV-unexposed infants in the CD56^dim^ subset (*p* = 0.005). Frequencies of CD38^bright^HLA-DR+ cells were highly variable (Figure 4), and we did not find a significant effect of HIV status or time in either the CD56^bright^, CD56+CD16+ or CD56-CD16+ subsets (Figure 4C; *p* > 0.05 for each comparison of mean difference and slope).

### Apoptosis-vulnerability during acute HIV infection

**Figure 5:**
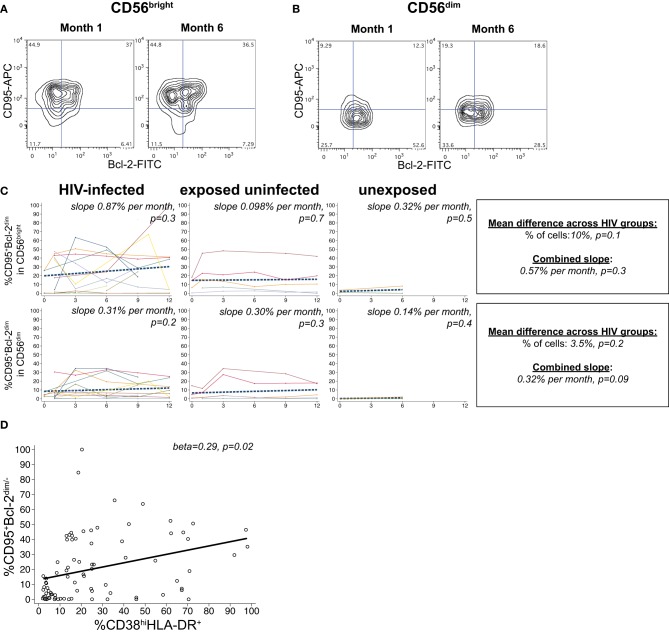
**CD95+Bcl-2^dim^ NK cells.** Representative flow cytometry contour plots from NK cell subsets showing CD95 and Bcl-2 staining in **(A)** CD56^bright^ and **(B)** CD56^dim^ subsets at 1 and 6 months of age an HIV-infected infant (B1-093). **(C)** Plots show individual infant trajectories as connected lines of different colors, with overlaid linear mixed models (LMM). LMM were used to determine whether slope (change in % of cells/month) was affected by HIV infection status; where slopes are not different, the slope representing the combined data of all infants is shown to the box on the right. The mean difference across HIV groups is derived from the beta coefficient for [HIV status] as described in the Materials and Methods, and represents the % increase in cells per HIV category from HIV-unexposed to HIV-exposed uninfected to HIV-infected. Slopes for each HIV infection group are also presented separately within each graph. **(D)** LMM model shows correlation between percentages of CD38^bright^ HLA-DR+ cells and CD95+Bcl-2^dim^ cells in the CD56^bright^ subset, using data from all infants.

A substantial frequency of apoptosis-vulnerable CD95^+^Bcl-2^dim^ cells was observed within the CD56^bright^ subset, with a lower percentage in the CD56^dim^ population (Figures 5A,B). We did not detect a significant effect of HIV infection status on the percentage of vulnerable NK in either the CD56^bright^ or CD56^dim^ subset (*p* > 0.05 for each, Figure 5C). Although percentages of apoptosis-vulnerable cells increased in many infants, this did not reach statistical significance in either the CD56^bright^ or CD56^dim^ subset (*p* > 0.05 for each). In HIV-infected infants, the percentage of apoptosis-vulnerable cells in the CD56^bright^ subset was strongly correlated with the percentage of CD38^bright^HLA-DR^+^ cells (beta = 0.29, *p* = 0.02; Figure 5D), suggesting activation-induced cell death (AICD) may account for the high level of vulnerable cells in the CD56^bright^ subset.

### NK cell differentiation during acute HIV infection

**Figure 6:**
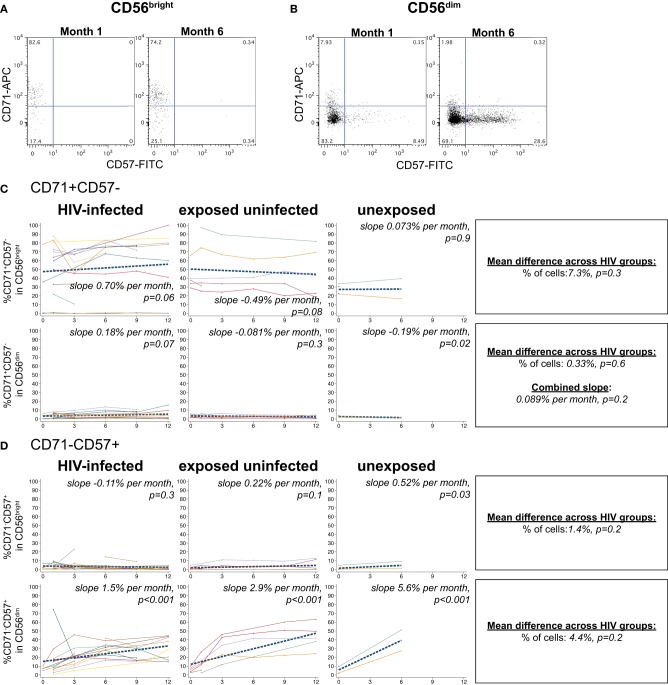
**CD71 and CD57 expression on NK cells.** Representative flow cytometry plots from NK cell subsets showing CD71 and CD57 staining in **(A)** CD56^bright^ and **(B)** CD56^dim^ subsets at 1 and 6 months of age an HIV-infected infant (B1-093). **(C** and **D)** Plots show individual infant trajectories as connected lines of different colors, with overlaid linear mixed models (LMM). LMM were used to determine whether slope (change in % of cells/month) was affected by HIV infection status; where slopes are not different, the slope representing the combined data of all infants is shown to the box on the right. The mean difference across HIV groups is derived from the beta coefficient for [HIV status] as described in the Materials and Methods, and represents the % increase in cells per HIV category from HIV-unexposed to HIV-exposed uninfected to HIV-infected. Slopes for each HIV infection group are also presented separately within each graph.

The transferrin receptor, CD71, regulates intracellular iron levels and is undetectable on resting lymphocytes but upregulated during their proliferation (Tormey et al., 1972; Dillner-Centerlind et al., 1979; Trowbridge and Omary, 1981). NK cells expressed substantial levels of the transferrin receptor, CD71, indicating recent proliferation (Figure 6). NK cells expressed either CD71 or the differentiation marker CD57 exclusively (Figures 6A,B). HIV-1 status did not have a significant effect on the percentage of CD71+CD57– cells in either the CD56^bright^ or CD56^dim^ subset (*p* > 0.05 for each). However, HIV status did have an effect on the change in CD71+CD57- cells over time in the CD56^bright^ subset (*p* = 0.03 for comparison of slopes by HIV category); we observed a trend for increasing frequencies of CD71+CD57- cells in the HIV-infected infants (+0.70% per month, *p* = 0.06), slowly decreasing frequencies of CD71+CD57- cells in HIV-exposed uninfected (–0.49% per month, *p* = 0.08), and no change among HIV-unexposed (+0.073% per month, *p* = 0.9) (Figure 6C).

In all infant groups, the percentage of differentiated CD71-CD57+ cells in the CD56^dim^ subset increased dramatically, consistent with CD56^dim^ NK cells serving as terminal effector cells (Figure 6D). The HIV-unexposed infants had the most rapid increase, gaining 5.6% per month (*p* < 0.001), followed by the HIV-exposed uninfected (+2.9% per month, *p* < 0.001) and the HIV-infected infants (+1.5% per month, *p* < 0.001). Although HIV-1 status did not have a significant impact on the percentage of CD71-CD57+ cells from the CD56^dim^ subset (*p* = 0.2), HIV-1 status did impact the rate of accumulation of these cells (comparison of slope by HIV status *p* = 0.004).

### Perforin levels decline in response to age and HIV infection

**Figure 7:**
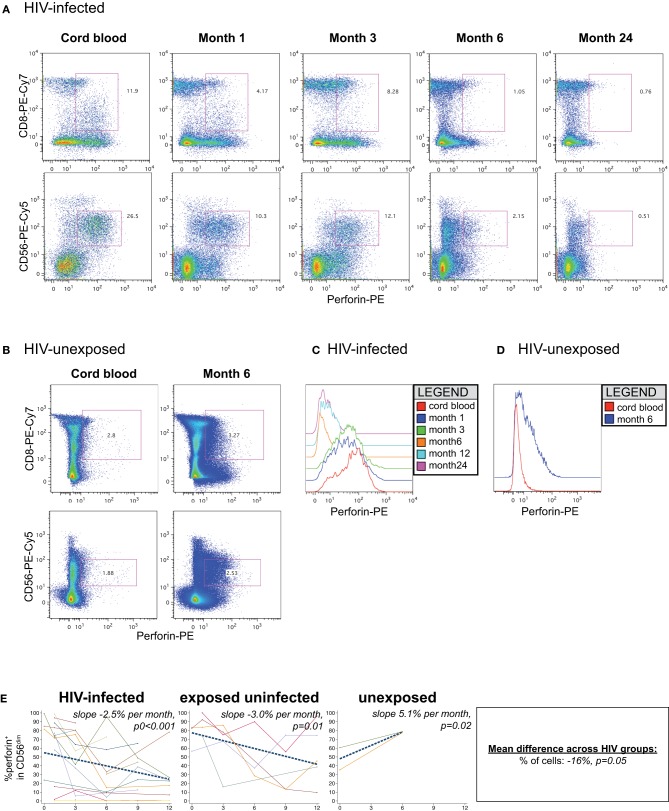
**Perforin content of NK cells.** Representative flow cytometry plots gated on lymphocytes show perforin content of CD8 and NK cells over time in an **(A)** HIV-infected (B1-093) and **(B)** HIV-unexposed infant (B1-119). Histograms show perforin levels at serial time-points in an **(C)** HIV-infected and **(D)** HIV-unexposed infant, gated on the CD56^dim^ subset. **(E)** Plots show individual infant trajectories as connected lines of different colors, with overlaid linear mixed models (LMM). LMM were used to determine whether slope (change in % of cells/month) was affected by HIV infection status. The mean difference across HIV groups is derived from the beta coefficient for [HIV status] as described in the Materials and Methods, and represents the % increase in cells per HIV category from HIV-unexposed to HIV-exposed uninfected to HIV-infected. Slopes for each HIV infection group are also presented separately within each graph.

In cord blood lymphocytes, perforin was preferentially expressed in CD56^dim^ NK cells, with lower levels observed in CD8+ T lymphocytes, as expected (Figures 7A,B). In HIV-infected infants, both the level of perforin (Figures 7C,D) and the percentage (Figure 7E) of perforin-positive cells declined dramatically over time in the CD56^dim^ subset. HIV-infected infants lost approximately 2.5% of perforin-positive cells per month (*p* < 0.001). The HIV-exposed uninfected infants also experienced a decline in the percentage of perforin-positive cells over time (−3.0% per month, *p* = 0.01). In contrast, the HIV-unexposed infants experienced an increase in perforin-positive cells over time (Figures 7B,E; +5.1% per month, *p* = 0.02), consistent with prior results indicating acquisition of perforin over time (Luevano et al., 2012).

### Impact of HIV viral load on NK subsets and phenotype

Finally, we examined the associations between viral load and NK cell subset and phenotype in the HIV-infected infants. HIV-1 RNA viral load was not significantly associated with either the total NK cell percentage (*p* = 0.7), or the percentage of CD56^bright^ (*p* = 0.7), CD56+CD16+ (*p* = 0.5) or CD56-CD16+ (*p* = 0.4) cells.

For each 1-log_10_ increase in HIV-1 viral load, we observed a 4.3% increase in the percentage of CD38^bright^HLA-DR+ cells in the CD56^bright^ subset (Table 4, *p* = 0.03). HIV-1 viral load was negatively associated with the percentage of CD71^+^CD57^−^ cells in the CD56^bright^ subset (beta coefficent = −4.5, *p* = 0.02). There were no significant associations between the percentage of early activated, apoptosis vulnerable, terminally differentiated, or potentially cytotoxic NK cells and HIV-1 viral load in infected infants.

**Table 4:** **Associations between HIV-1 RNA viral load and NK cell phenotype**.

**Phenotype**	**Subset**	**Mean increase in % of cells per 1-log increase in HIV-1 RNA viral load^a^**	***P*-value**
Early activated	CD56^bright^	0.80	0.6
(CD38^bright^CD69^+^)	CD56^dim^	0.71	0.6
Activated	CD56^bright^	4.3	0.03
(CD38^bright^HLA-DR^+^)	CD56^dim^	0.14	0.9
Vulnerable	CD56^bright^	1.2	0.7
(CD95^+^Bcl-2^dim/-^)	CD56^dim^	1.6	0.3
Proliferating	CD56^bright^	−4.5	0.02
(CD71^+^CD57^−^)	CD56^dim^	−0.40	0.3
Terminally differentiated	CD56^bright^	0.74	0.3
(CD71^−^CD57^+^)	CD56^dim^	+3.4	0.1
Potentially cytolytic	CD56^bright^	−6.6	0.1
(perforin+)	CD56^dim^	−6.0	0.1

a *Beta coefficient for association between HIV-1 viral load and NK cell phenotype from linear mixed-effects models with HIV-1 RNA viral load included in a model with age*.

## Discussion

Here we report that infant HIV infection is associated with alterations in NK cell subsets, activation, and cytolytic potential that could contribute to their poor control of HIV infection over the first year of life. Additionally, the HIV-exposed uninfected group exhibited a phenotype that was intermediate between HIV-infected and HIV-unexposed infants in terms of activation and perforin expression. This “intermediate” phenotype of HIV-exposed uninfected infants could be indicative of the detrimental effects of chronic exposure to HIV, or could reflect a phenotype more protective from HIV acquisition, in which relatively blunted levels of activation, decreased proliferation, and more rapidly maturing NK cells could contribute to resistance to HIV acquisition.

Several studies have indicated that NK cells play an important role in the containment of HIV replication during primary infection in adults (Fauci et al., 2005; Alter and Altfeld, 2009). However, infant NK cells appear to be compromised in their cytolytic capacity (Phillips et al., 1992; Bradstock et al., 1993; Gaddy and Broxmeyer, 1997; Tanaka et al., 2003; Luevano et al., 2012), and though they can suppress HIV replication *in vitro* by secreting antiviral cytokines (Bernstein et al., 2004), it is not clear whether these changes in NK cell function could contribute to poor viral control. Although we were unable to directly assess NK cell functions in this study, we hoped to gain insight the effects of primary HIV infection and exposure on NK cell phenotype in order to better understand the dynamic interplay between NK cells and virus.

In adults, acute HIV infection is associated with a transient expansion in CD56+CD16+ NK cells that is followed by a decline in total NK cell numbers and the development of an anergic CD56-CD16+ NK cell population (Alter et al., 2005; Björkström et al., 2010; Hong et al., 2010a). Since these alterations in NK cell subset distribution and function are likely related to active viral replication, the increased viral replication typical of infant HIV infection could accelerate development of NK cell dysfunction and contribute to poor control over viral replication. As in adults, we observed a decline in total NK cell percent in HIV-infected infants over the first year of life (Azzoni et al., 2002; Alter et al., 2005; Mantegani et al., 2010). However, the effect of HIV-infection on NK subset distributions is less clear since CD56-CD16+ NK cells were found in cord blood all infants at birth (Bradstock et al., 1993; Gaddy et al., 1995). Thus, the presence of such cells could be a “normal” phenomenon, which may be exacerbated by the effects of HIV infection. Consistent with this hypothesis, we observed an increase in the proportion of CD56-CD16+ NK cells over the first year of life in all infant groups. It is possible that cytokine milieu required for maturation of CD56-CD16+ neonatal NK cells may not fully develop until after the first year of life, and that HIV infection further compromises production of IL-2, IL-12, and/or IL-15 (Gaddy and Broxmeyer, 1997; Yokoyama et al., 2004; Luevano et al., 2012). In addition, in HIV-infected babies, active viral replication could induce CD56^dim^ cells to downregulate CD56 (Alter et al., 2005; Björkström et al., 2010). The persistence and functions of this atypical NK cell population in infants warrants further evaluation.

Among the CD56+ subpopulations of NK cells, we observed that CD56^bright^ and CD56^dim^ populations had relatively stable characteristics that did not appear to be modified by HIV infection or exposure. While both populations expressed markers of activation, CD56^bright^ cells were more likely to proliferate and had more cells vulnerable to apoptosis. In contrast, consistent with their role as effectors, the CD56^dim^ cells were more likely to be terminally differentiated (CD71-CD57+) and express perforin. These data highlight the dynamic status of the NK cell population in the first year of life, in which the CD56^bright^ cells may be actively proliferating to fill the CD56^dim^ effector cell pool. The CD56^bright^ cells appeared to be more sensitive to perturbation caused by HIV, consistent with results in adults (Mantegani et al., 2010). Since activation was strongly correlated with vulnerability to apoptosis, CD56^bright^ cells may also be more susceptible to AICD.

Infant HIV infection was associated with an altered phenotype within specific NK subsets. There appeared to be a stepwise effect of HIV infection and exposure on CD69 expression on NK cells, with HIV-infected infants having the highest expression of this marker on both CD56^dim^ and CD56^bright^ NK cells, followed by HIV-exposed uninfected infants and then HIV-unexposed infants. In fact, although we did not have sufficient power to observe significant differences in many markers, HIV-exposed uninfected babies also appeared to be intermediate in phenotype between HIV-infected and HIV-unexposed babies in terms of perforin expression, activation status, and percentage of NK cell subsets. These results are consistent with prior studies in which lymphocytes from HIV-infected and exposed babies had increased susceptibility to apoptosis in comparison to unexposed babies (Economides et al., 1998). In addition, CD4+ T cells from HIV-exposed uninfected infants became more activated yet secreted less IL-2 than HIV-unexposed infants (Rich et al., 1997). HIV exposure is associated with alterations in cytokine production by cord blood mononuclear cells (Chougnet et al., 2000; Kuhn et al., 2001), which could modulate NK cell activity in HIV-infected and HIV-exposed uninfected babies.

Acute HIV infection and exposure was associated with dramatic alterations in the development of mature, functionally competent NK cells, as has previously been observed in adults (Hong et al., 2010b). In all infant groups, the percentage of CD57 cells increased rapidly during the first year of life; however, HIV infection and exposure were associated with a slower increase in fully differentiated, CD57+ NK cells, even though there were not significant differences in the percentage of these cells between groups. We suspect that the accumulation of CD57+ NK cells may be counterbalanced by greater overall rates of AICD in HIV-infected and HIV-exposed uninfected infants, though in our small study we had limited power to evaluate this. We also observed an increase in CD71 levels only in HIV-infected infants; this could suggest that NK cell proliferation is increased in this population, possibly as a response to persistent antigenic stimulation.

The most dramatic effect of HIV exposure and infection on infant NK cells was observed in the expression of perforin, a marker of cytolytic potential. As previously described, only a subset of CD56^dim^ NK cells in cord blood expressed perforin (Gaddy et al., 1995; Gaddy and Broxmeyer, 1997). As expected, the expression of perforin increased in CD56^dim^ cells in healthy, HIV-unexposed babies. However, HIV-infection, and less dramatically, exposure in uninfected babies, was associated with a decrease in expression of perforin over time. Similar results of low perforin expression during chronic HIV infection have been noted in chronically HIV-infected adults (Alter et al., 2006; Qi et al., 2006). This low perforin expression could reflect the fact that the NK cells are actively degranulating in response to HIV infection or that the aberrant inflammatory environment during HIV-1 infection is deficient in cytokines such as IL-15 that are required for development of fully mature, perforin-expressing NK cells. Both mechanisms could contribute to the dramatic decline in perforin expression in HIV-infected infants. However, HIV-exposed uninfected infants also have low perforin levels that decline with age. If this is a phenotype reflects a “protective phenotype,” this could mean that non-cytolytic methods, such as cytokine secretion, play a more important role in preventing HIV-1 acquisition than cytolysis. However, the decline in perforin could also reflect the detrimental effects of chronic exposure to HIV-1 and possibly other pathogens from the HIV-infected mother. Regardless of the mechanisms, the poor expression of perforin could compromise cytolytic capacity of NK cells, and contribute to the poor ability of HIV-infected and, to a lesser extent, HIV-exposed uninfected infants to control viral infection. Interestingly, Ballan et al. found that HIV-infected children on ART had similar levels of perforin as HIV-exposed uninfected children (Ballan et al., 2007). As this study was of older children who received ART, it was not clear whether provision of ART was associated with recovery of perforin expression. Furthermore, this study did not include a HIV-unexposed cohort, so it is not clear whether perforin levels were low in both the HIV-infected/ART and HIV-exposed uninfected children relative to healthy, unexposed children. Together, our data indicate that HIV exposure *in utero* and the maternal environment can lead to relatively long-lasting effects on infant immunity.

In HIV-infected infants, the level of viral replication was not significantly associated with the total NK cell percentage or subset distribution. This is somewhat distinct from the findings in adults, in which higher levels of replication as associated with increased frequencies of CD56-CD16+ NK cells (Alter and Altfeld, 2009). However, the viral load was positively associated with the percentage of activated CD38^bright^ HLA-DR+ NK cells and inversely associated with the percentage of CD71+CD57- NK cells. These data suggest that high levels of HIV viral replication during acute HIV infection may result in persistent activation and cycling of NK cells, similar to what we have previously observed in CD8 T cells (Slyker et al., 2011).

Our study has several strengths, and also some limitations. To our knowledge, this study represents the first longitudinal evaluation of HIV-infected and exposed infants during the first year of life; with longitudinal follow-up, we were thus able to avoid survival bias present in previous studies which evaluated HIV infection in older children (Azzoni et al., 2005; Ballan et al., 2007). As myeloid lineage markers were not used, it is possible that some of the CD56-CD16+ were small myeloid lineage cells. Since this was a retrospective study, we used frozen cells, which could potentially have skewed our phenotypic data, due to preferential loss of some cell subsets (particularly activated and apoptotic), and were unable to perform direct assessments of NK cell functions such as cytolytic activity. Due to limitations in our staining panels, we were unable to examine expression some markers previously shown to be associated with functional alterations, such as expression of CD161 on NK T cells (Snyder-Cappione et al., 2009). Our limited sample size, particularly for HIV-exposed uninfected and HIV-unexposed babies, decreased statistical power to detect significant differences between groups, so we interpret these results cautiously; indeed, with a larger sample size additional effects of HIV-infection, or HIV-exposure are likely to be found. Despite this limitation, we consistently observed stepwise effects on NK cell phenotype, with the HIV-exposed uninfected babies intermediate between HIV-infected and HIV-unexposed babies. Other studies of HIV infection and exposure have confirmed that HIV-exposed uninfected babies have alterations in cytokine production that could modulate NK cell activity (Rich et al., 1997; Chougnet et al., 2000; Kuhn et al., 2001). Finally, NK cell distribution is likely altered by CMV infection, which is highly prevalent in this population (Slyker et al., 2009). However, although nearly all infants acquired CMV infection by 3 months of age, we did not note changes in NK cell phenotype concurrent with acute CMV infection (data not shown).

In conclusion, in this well-characterized cohort we were able to evaluate longitudinal effects of HIV exposure and infection on NK cells in infants. HIV infection was associated with decreased total NK cell numbers, increased in differentiation, and a significant decline in perforin expression over time, which could functionally compromise infant NK cells. We observed a similar, though muted pattern in the NK cells of HIV-exposed uninfected infants. Together these data suggest that HIV-induced alterations in the NK cells may contribute to both poor viral control in HIV-infected infants and altered susceptibility to infection in HIV-exposed uninfected infants.

### Conflict of interest statement

The authors declare that the research was conducted in the absence of any commercial or financial relationships that could be construed as a potential conflict of interest.
